# Determination of serum lipid profile in patients with diabetic macular edema that referred to Shahid Beheshti and Ayatollah Rouhani Hospitals, Babol during 2011-2012

**Published:** 2015

**Authors:** Seyed Ahmad Rasoulinejad, Habib-Ollah Iri

**Affiliations:** 1 Babol University of Medical Sciences, Babol, Mazandaran, Iran.; 2 Department of Ophtalmology, Babol University of Medical Sciences, Babol, Iran.

**Keywords:** Diabetes, Diabetic retinopathy, Macular edema, Lipid profile, Cholesterol.

## Abstract

**Background:**

Diabetes is a common metabolic disorder leading to the development of many complications, among which diabetic retinopathy and macular edema are the most significant. These complications can contribute to blindness if not diagnosed or treated properly, and several studies have been conducted to evaluate the methods for the prevention or slowing down their progression. Therefore, serum lipids, apparently play an effective role in the creation and acceleration of macular edema, we therefore determined the relationship of serum lipid level in patients with diabetic macular edema in the present study.

**Methods:**

180 participants were selected from patients with the definite diagnosis of diabetes referred to the eye clinic of Shahid Beheshti and Ayatollah Rouhani Hospitals of Babol during 2011-2012, the patients with a history of taking lipid –lowering drugs and hypertension were excluded from the study. The study data were provided from the medical records of each patients. SPSS Version 18 was used for analyses.

**Results:**

In the present investigation, the mean age of participants was 53.22±with the age range of 18-77 years. Ninety patients with diabetic retinopathy and macular edema were compared with ninety patients with diabetic retinopathy without macular edema (control group) were compared. There was a significant difference in serum cholesterol and LDL-cholesterol between patients and groups (p<0.000).

**Conclusion:**

The results of this study indicate that high serum cholesterol and LDL-cholesterol is associated with severity of diabetic retinopathy particularly with macular edema

The global prevalence of diabetes mellitus has dramatically increased during the past two decades and has reached from approximately 30 million cases in 1985 to 177 million in 2000. If the situation continues the same way, more than 360 million people will suffer from diabetes by 2030, and even some third world countries such as India will become the world diabetes center by this year (1-3). Diabetes induced systemic complications are influential on individuals as well as the society, since they affect diabetic patients in the most important and useful years of their productivity in the community (4, 5). Diabetes ocular complications (ophthalmologic) including retinopathy, macular edema, and so on are among the most important complications attracting great attention.

Diabetic retinopathy is classified into two stages: early stage (NPDR; non proliferative diabetic retinopathy) and advanced stage (PDR; proliferative diabetic retinopathy). People with diabetic retinopathy may lose their vision under the influence of one of the following reasons:

-macular edema (capillary leakage), macular ischemia (capillary blockage), and post-ischemic neovascularization (6, 7). 

Diabetic macular edema (DME) is a serious visual effect caused by abnormal permeability of retinal vessels in patients with diabetic retinopathy (7). Overall, macular edema is a major diabetes complication in type I and type II diabetic patients. Abnormal changes in the eye capillaries and capillary leakage lead to diabetic macular edema and possible blindness if not treated (8). The ETDRS has defined clinically significant macular edema (CSME) as herein below:

1-thickening of the retina at or within 500 microns of the center of the macula 

2-hard exudates at or within 500 microns of the center of the macula, if associated with thickening of the adjacent retina 

3-a zone or zones of retinal thickening one disc area or larger, any part of which is within one disc diameter of the center of the macula (7). 

Although the actual percentage of blindness cases caused by DME is still unknown in diabetic patients, the negative effect of DME on vision has always been a serious concern for diabetic population. DME patients are consuming large resources of health care system, and their expenditure is higher than those without complications of diabetic retinopathy (8). DME is manifested as increased focal or diffuse retinal thickness with or without retinal exudates; focal edema may be associated to rings of hard exudates derived from plasma lipoproteins which appear to have been caused by microaneurysm. Absorption of liquid components results in the deposition of lipid debris, which often occurs in internal and external plexiform layers and sometimes below the sensory retina. 

These remains are white to yellow deposits called hard exudates. As the density of these hard exudates increases, they tend to migrate towards the foveal center where their deposition predisposes to the development of sub foveal fibrosis leading to irreversible visual loss. ETDRS has demonstrated an independent adverse effect of these retinal hard exudates on patients visual (VA) (2, 9,10). 

Diffuse macular edema is accompanied with extensive abnormalities of retinal capillaries, resulting in diffuse leakage from large rupture of retina – blood barrier and is often associated with cystoid macular edema. In other words, in focal macular edema, spots of focal capillary leakage or microaneurysm exist in fluorescein angiography, while the disk does not contain obvious marginal leakage areas in diffuse type (7).

Various factors are involved in the incidence and severity of diabetic macular edema such as the onset of diabetes (chronic hyperglycemia and diabetes duration), Poor glycemic control, increased HbA1C level, hypertension, and hyperlipidemia (6) 

Generally, DME treatments include the following: 1. Change in lifestyle, 2. Laser, 3. Medical treatment, and 4. surgical treatment.

Serum lipids, which apparently play an effective role in the creation and acceleration of macular edema; we therefore determined the relationship of serum lipids level in patient with diabetic macular edema in the present study.

## Methods

180 participants selected from patients with definite diagnosis of diabetes referred to the eye clinic of Shahid Beheshti and Ayatollah Rouhani Hospitals, Babol during 2011-2012, the patients with a history of taking lipid –lowering drugs and hypertension were excluded from the study. The study information consisting of age, sex, diabetes duration, HbA_1_C level, FBS, serum level of VLDL, LDL, HDL, TG, and cholesterol were measured and recorded in the files and prepared for each patient. Based on macular and retinal examination, the subjects were divided into three categories ;a) Control group: 

1. Diabetic patients with retinopathy but without macular edema, 90 patients 

b) Case group: 2. Diabetic patients with retinopathy along with mild macular edema, 45 patients 3. Diabetic patients with severe macular edema and plaque – like hard exudates, 45 patients 

Data were collected, coded and then transferred to SPSS_18_ statistical software and were described using tables and figures after analysis. ANOVA and Tukey’s posttest were used for parametric data and p<0.05 was considered as a statistically significant level.

## Results

Parameters studied included age range and mean age of participants, number of patients in each group with separation based on gender, duration, of diabetes and serum level of HbA1C, FBS, cholesterol, TG, LDL,VLDL, and HDL. The mean diabetes duration was as follows for each group:

-Group 1: 9.82±6.58 (CI=8.47-11.16)

-Group 2: 15.17±6.92 (CI=13.01-1732)

-Group 3:13: 38±6.35 (CI=10.55-14.22)

There was a significant difference between control and vehicle –control groups (p=0.000). In the present investigation, the mean age of participants was 53.22± with the age range of 18-77 years. Age information of ten study subjects was described in details, in table 1 with separation based on the study groups. 

The comparison results of variable based on groups of disorders are demonstrated in table 2.

**Table 1:** Age distribution of diabetic patients participated in the study with separation based on groups of disorder

**Group**	**Mean±SD**	**CI95% **	**Range**
1	52.34±9.94	50.30±54.38	18-74
2	51.95±7.78	49.53±54.38	35-77
3	56.04±8.68	53.52±58.56	31-74
Total	53.22±9.27	51.87±54.47	18-77

1: Diabetic retinopathy without macular edema

2: Mild macular edema

3: Macular edema + hard exudates Plaque

**Table 2:** Comparison of the variables value with separation based on groups of disorder

	**1** **Mean±SD**	**2** **Mean±SD**	**3** **Mean±SD**	**P. Value**
HbA1C	9.53±1.848	9.23±1.505	9.77±1.774	423.0
FBS	208.03±74.0.75	217.77±63.46	215.63±77.118	0.729
Cholesterol	202.95±53.402	204.17±52.213	228.74±113.201	0135
TG	212.74±227.911	187.43±109.448	218.28±238.741	0.779
VLDL	37.09±22.396	36.85±20.238	33.51±15.127	0.625
LDL	113.39±38.487	113.08±42.089	132.33±53.756	0.049
HDL	44.25±15.586	47.32±22.920	48.49±11.009	0.323

## Discussion

 In the present survey implemented on diabetic patients with retinopathy, the total number of participants was 180 patients divided into the following groups: 

1. Patients with diabetic retinopathy without macular edema

2. Patients with mild macular edema

3. Patients with macular edema along with hard exudates plaque 

In this study, serum levels of FBS, HbA_1_ C, TG, Cholesterol, VLDL, LDL and HDL were evaluated for all the three groups. There was a significant correlation between control and vehicle control group in terms of diabetes duration and disorder severity (p=0.000), which is similar to the study of Ozera et al. in 2008 (11). In a study conducted in Australia, the incidence of retinal damage has been augmented with increasing duration of diabetes. In this investigation, it has been shown that the prevalence of DR has been less than 10% in patients with less than five years of diabetes onset to more than 50% in patients with 20 or more years of duration of diabetes (12). In a study by Er Chen et al. in 2010, the frequency of diabetic macular edema was strongly dependent on types and duration of diabetes as well as patient age at diagnosis (8); this issue can be justified regarding longer hyperglycemic conditions followed by increased duration of retinal vascular damage.

In recent decades, it has been determined that glycosylation and free radicals – induced oxidative stress during chronic hyperglycemia can eventuate in damage to cell components such as lipids, proteins, and DNA, leading to the development of complications (13-15). Moreover, high blood glucose for a long period of time can cause alterations in pericytes and basic membrane, contributing to endothelial barrier dysfunction (7).

No significant relationship was found between HbA1C level and severity of macular edema in the present study, although the mean HbA1C was higher in the third group than the other groups, the difference was not statistically meaningful (p>0.05). The mean HbA1C was 9.53±1.77 mg/dl, which is higher than normal, and can indicate lack of proper control on diabetes among these patients; this finding is in accordance with the results obtained by Ozera et al. in 2008 in TUKEY (11). 

In our study, no significant relationship was observed between FBS level and severity of macular edema, although FBS level was higher in the case group than the control. It was not statistically meaningful (p>0.05). The serum level of cholesterol was not remarkable, which is in agreement with Ozera et al’s study in 2008 (11). Similarly, in a study at Harvard University by Biljana Milianovic et al., no significant correlation was found between serum cholesterol level and progression of retinopathy after demonstrating HbA1C values (16). Likewise, although the mean serum triglyceride was higher in the third group in our study, the association was not statistically significant.

In this study, the mean serum HDL as well as VLDL level revealed no meaningful correlation between three groups, about which finding on VLDL level is in consistence with Ozera’s study (11) and also in the work of Nil Irem et al. on 54 patients divided into two groups (n=27), one with hard exudates and the other one without hard exudates, between which no remarkable difference was detected for the level of triglycerides, VLDL and HDL (17).

In addition, in a study by Barbara E.K Klein et al. in the United States, no significant relation was displayed between serum cholesterol and HDL levels with the progression of retinopathy or macular edema (18).

In another investigation by Ming et al. in 2009 on 51 male patients, lipid profiles of the study participants were measured, and no correlation was obtained between the serum level of HDL and diabetic retinopathy. In the present study, the mean LDL serum level was 118.8 which was higher in the third group (patients with macular edema and hard exudates) than other groups, and the difference was statistically significant (p-value <0.05), similar to the findings of Raman and Rani, in 2010, on 1714 patients, among which the LDL serum level was higher in patients with diabetic macular edema (19).

 In a 15-year study by Romer et al., in 2006, for the evaluation of macular edema and its possible risk factors on diabetic patients without retinopathy and nephropathy, it has been suggested that monitoring LDL levels decrease progression of macular edema (20). Roy and Klein had exhibited that proteinuria, male gender, and higher levels of LDL and duration of diabetes are significantly and independently associated with the severity of retinal hard exudates (21). Besides, in 2008, Sanchez et al. demonstrated that CSME is in association with older age, proteinuria, HbA1C, and increased level of LDL and cholesterol (22). In a series of studies, it was found that LDL has a toxic effect on pericytes and retinal capillaries (10).

Diabetes as a major cause of diabetic retinopathy and blindness in adults leads to an increase in LDL oxidation, and modified LDL (oxidized or glycosylated) reduces gene expression in pericytes (13), and mitochondrial dysfunction and pericytes apoptosis occur following these change (23). Furthermore, in animal models (bovine), oxidized LDL brings reduction in pericytes lifetime and destruction of endothelial cells (24). Despite the results of our study, other investigations were conducted, in which significant correlation had been found between the levels of serum lipids (cholesterol) and retinal hard exudates. For instance, in a study by Lyons et al. in 2004, to determine the relationship between diabetic retinopathy and different subgroup of serum lipids on 440 females and 548 males, it was announced that the intensity of retinopathy has direct association with elevated triglyceride and LDL, but had adverse relations with high – density lipoprotein (HDL) (24).

A survey was conducted to evaluate the effective risk factors in the creation of hard exudates in type II diabetic patients in Indian population in 2010, in which 180 patients (116 males and 64 females with mean age of 55.6years) with type II diabetes along with NPDR and or CSME were examined. Participants were divided into three groups: 1. Patients without or with mild hard exudates 2.Patients with hard exudates 3.Patients with significant hard exudates. In this study, serum cholesterol and LDL levels were introduced as independent risk factors in the creation of diabetic hard exudates (2).

In Conclusion this study, it has been demonstrated that serum level of LDL is associated with the severity of diabetic macular edema and hard exudates.

## Notes

**Funding:** Self-funded.
